# The Antidiabetic Activity of Wild-Growing and Cultivated Medicinal Plants Used in Romania for Diabetes Mellitus Management: A Phytochemical and Pharmacological Review

**DOI:** 10.3390/ph18071035

**Published:** 2025-07-11

**Authors:** Diana Maria Trasca, Dalia Dop, George-Alin Stoica, Niculescu Stefan Adrian, Niculescu Elena Carmen, Renata Maria Văruț, Cristina Elena Singer

**Affiliations:** 1 Department of Internal Medicine, University of Medicine and Pharmacy of Craiova, 200349 Craiova, Romania; diana.trasca@umfcv.ro; 2 Department of Mother and Baby, University of Medicine and Pharmacy of Craiova, 200349 Craiova, Romania; dalia.dop@umfcv.ro (D.D.); cristina.singer@umfcv.ro (C.E.S.); 3 Department of Pediatric Surgery, Faculty of Medicine, University of Medicine and Pharmacy of Craiova, 200349 Craiova, Romania; 4 Department of Orthopedics, University of Medicine and Pharmacy Craiova, 200349 Craiova, Romania; niculescustefan94@gmail.com; 5 Research Methodology Department, Faculty of Pharmacy, University of Medicine and Pharmacy of Craiova, 200349 Craiova, Romania

**Keywords:** diabetes mellitus, medicinal plants, phytotherapy, hypoglycemic activity, Romanian ethnobotany

## Abstract

Diabetes mellitus is a chronic metabolic disease that has a significant impact on public health and is becoming more and more common worldwide. Although effective, conventional therapies are often limited by high cost, adverse effects, and issues with patient compliance. As a result, there is growing interest in complementary and alternative therapies. Medicinal plants have played an essential role in diabetes treatment, especially in regions such as Romania, where biodiversity is high and traditional knowledge is well preserved. The pathophysiology, risk factors, and worldwide burden of diabetes are examined in this review, with an emphasis on the traditional use of medicinal plants for glycemic control. A total of 47 plant species were identified based on ethnopharmacological records and recent biomedical research, including both native flora and widely cultivated species. The bioactive compounds identified, such as flavonoids, triterpenic saponins, polyphenols, and alkaloids, have hypoglycemic effects through diverse mechanisms, including β-cell regeneration, insulin-mimetic action, inhibition of α-glucosidase and α-amylase, and oxidative stress reduction. A systematic literature search was conducted, including in vitro, in vivo, and clinical studies relevant to antidiabetic activity. Among the species reviewed, *Urtica dioica*, *Silybum marianum*, and *Momordica charantia* exhibited the most promising antidiabetic activity based on both preclinical and clinical evidence. Despite promising preclinical results, clinical evidence remains limited, and variability in phytochemical content poses challenges to reproducibility. This review highlights the potential of Romanian medicinal flora as a source of adjunctive therapies in diabetes care and underscores the need for standardization and clinical validation.

## 1. Introduction

Diabetes mellitus (DM) ranks among the top global health burdens, trailing only cardiovascular and oncological diseases in severity and frequency. Its prevalence has soared, affecting about 6.6% of the global population, and healthcare systems allocate over 10% of resources to its management [1,2]. From 2000 to 2011, cases doubled from 171 million to 347 million, with projections exceeding 550 million by 2030 [3].

In Romania, type 1 DM incidence sits near 5 per 100,000 annually, while many more live in a prediabetic state that, without lifestyle and dietary intervention, will progress to overt diabetes [4]. The World Health Organization divides DM into type 1, type 2, other specific forms, and gestational diabetes, with type 1 and type 2 being the most prevalent. This spectrum, from normoglycemia through insulin dependence, aids accurate staging and guides therapy. Type 1 diabetes is characterized by autoimmune destruction of pancreatic β-cells, resulting in absolute insulin deficiency, while type 2 diabetes is marked by insulin resistance and a relative insulin deficiency due to impaired β-cell function. Insulin resistance refers to reduced sensitivity of peripheral tissues to the effects of insulin, leading to compensatory hyperinsulinemia and eventual glucose intolerance. One key mechanism targeted by both synthetic and natural therapies is α-glucosidase inhibition, which slows the intestinal breakdown of carbohydrates, thereby reducing postprandial blood glucose spikes. Etiologically, DM may be primary (idiopathic) or secondary to identifiable conditions such as chronic pancreatitis, hormonal disorders (e.g., Cushing disease), certain medications or toxins, and genetic syndromes [5,6].

Type 1 DM stems from an autoimmune attack; T lymphocytes destroy pancreatic β-cells, leading to rapid-onset symptoms: polyuria, polydipsia, polyphagia, weight loss, and fatigue. Without prompt insulin therapy, patients risk ketoacidosis and coma [5]. Both genetic factors (diabetogenic HLA haplotypes) and environmental triggers (viral infections, mumps, Coxsackie B4, rubella, CMV, EBV, and early dietary exposures like cow milk proteins, nitrosamines, and caffeine) contribute to disease onset [7].

Type 2 DM evolves insidiously from insulin resistance and impaired secretion. It often goes undetected for years. Key risk factors include sedentary lifestyle, excessive caloric intake, dyslipidemia, hypertension, and obesity. In obesity, enlarged adipocytes and infiltrating immune cells release pro-inflammatory adipokines, worsening insulin resistance and promoting endothelial dysfunction [8,9].

Persistent hyperglycemia fosters oxidative stress—an imbalance favoring reactive oxygen species (ROS) over antioxidants (glutathione, catalase, and superoxide dismutase), damaging cells and driving complications such as retinopathy, neuropathy, nephropathy, atherosclerosis, and heightened risks of myocardial infarction and stroke [10]. Consequently, diabetic individuals face markedly increased cardiovascular, renal, and ocular morbidity. Neither a cure nor fully substitutive insulin exists. Management centers on staged interventions: lifestyle modification (low glycemic index, diet, and exercise), phytotherapy, oral antidiabetics, and insulin injections [11].

This review focuses on medicinal plants that are either native to Romania or widely used in Romanian ethnomedicine, including both indigenous wild flora and cultivated or imported species with documented antidiabetic properties. Romania possesses a unique cultural and ethnobotanical heritage, with centuries-old traditions of using medicinal plants for managing chronic diseases such as diabetes. Rural and urban communities alike have preserved knowledge regarding the preparation and therapeutic use of indigenous and introduced species, making Romania a particularly rich resource for the study of plant-based antidiabetic interventions.

Phytotherapy, used since antiquity, employs plant-based preparations (infusions, decoctions, and tinctures) rich in bioactive compounds. Its benefits include the synergistic effects of phytocomplexes and a favorable side-effect profile [12]. Research has linked hypoglycemic activity to constituents such as polyphenolic acids, anthocyanosides, carotenoids, essential oils, triterpenes, tannins, and flavonoids. The mechanisms include β-cell regeneration, insulin-mimetic action, reduction in intestinal glucose uptake, suppression of gluconeogenesis, enhancement in peripheral glucose utilization, and improvement in insulin sensitivity [13].

Animal studies, often using streptozotocin or alloxan to induce diabetes, have clarified these mechanisms and supported the traditional use of many plants for reducing hyperglycemia and oxidative stress [14].

## 2. Pathogenesis of Diabetes Mellitus

Type 1 diabetes (T1DM) arises when autoimmune Th1-polarized CD4^+^ and CD8^+^ T cells (and macrophages) attack pancreatic β-cells in the islets of Langerhans, often despite normal B-cell function. This breakdown in self-tolerance is linked to defective thymic deletion of high-affinity T cells and MHC gene variants. β-cell loss occurs via direct CD8^+^ cytotoxicity, cytokines (IL-1, IFN-γ, TNF-α), ROS, nitric oxide, or Fas/FasL interactions [15].

Three autoantibodies, ICA-512, anti-GAD65, and insulin autoantibodies (IAA), appear years before symptoms. A single autoantibody carries a ~20% risk of clinical T1DM; two or more raise the risk to above 75% [16]. Familial risk hinges on HLA class II alleles: DRB103/04 and DQB10302 confer ~25% risk, doubling to ~50% if multiple relatives are affected [17,18,19]. Intriguingly, some carriers of the “protective” DQA1-DQB1*0602 haplotype still develop T1DM—suggesting delayed onset rather than absolute protection—and the precise pathogenic role of autoantibodies remains uncertain [20]. Early in T1DM, insulin sensitivity is preserved, but endogenous secretion plummets by diagnosis (low C-peptide), necessitating lifelong insulin replacement to avert ketoacidosis and other acute crises [21,22].

Type 2 diabetes (T2DM) reflects peripheral insulin resistance, driven by altered receptor signaling and eventual β-cell exhaustion, and a modest genetic predisposition (e.g., TCF7L2 variants increase risk ~1.5-fold) [23]. Contributing metabolic derangements include heightened adipocyte lipolysis, incretin resistance, hyperglucagonemia, fluid retention, and dysregulated central metabolic control.

Obesity is a pivotal, modifiable driver of T2DM. Hypertrophic white adipose tissue becomes inflamed; macrophage infiltration and excess TNF-α and IL-6 disrupt insulin signaling, while leptin overproduction further stimulates macrophages [24]. Conversely, adiponectin, normally abundant in adipose tissue and protective via the suppression of hepatic gluconeogenesis, the promotion of muscle fatty acid oxidation, and anti-inflammatory effects, is markedly reduced in obesity and diabetes, exacerbating insulin resistance and atherogenesis [25,26].

## 3. Causes and Risk Factors

The causes of diabetes mellitus remain only partially understood. The development of both major types of diabetes is closely associated with hereditary factors, particularly family history involving first-degree relatives (parents or siblings) diagnosed with the condition. Researchers have identified at least 18 genetic loci, designated IDDM1 through IDDM18, that are correlated with T1DM. The IDDM1 region includes the HLA genes, which regulate immune responses and encode proteins of the major histocompatibility complex. In addition to genetic background, phenotypic expression plays a significant role.

Currently, extensive genetic studies aim to define specific genetic profiles that predispose individuals to T2DM in various regions around the world. T2DM is considered a polygenic disorder, involving multiple genes located on different chromosomes that contribute to disease susceptibility. The analysis of genetic contributions is complicated by the fact that numerous environmental factors interact with these genes to trigger disease onset. Only a minority of T2DM cases are attributable to single-gene defects, one notable example being maturity-onset diabetes of the young (MODY). Our current understanding of the genetic basis of T2DM remains limited. Reflecting the complex network of physiological disturbances in T2DM, its genetic architecture involves a large number of susceptibility genes, each exerting a relatively small effect (Table 1).

Several studies suggest that vitamin D deficiency during childhood increases the risk of later developing T1DM. In Finland, a northern country with limited sunlight exposure and reduced cutaneous vitamin D synthesis, the incidence of T1DM is the highest in the world. Other research has identified early cow milk consumption as another predisposing factor for autoimmune diabetes. Bovine serum albumin can trigger the production of autoantibodies that, through molecular mimicry, target pancreatic β-cells.

Gluten and other cereal-derived proteins have also been implicated as potential antigens; introducing cereals into an infant’s diet before the age of 3 months may increase the risk of developing T1DM.

Additional contributors include metabolic stress and exposure to environmental toxins, both of which may predispose individuals to the development of T1DM [30].

For T2DM, the most significant modifiable risk factors include excessive body weight, primarily due to overeating and physical inactivity [31]. Another potentially important diabetogenic factor is psychological stress, especially crowding-related stress in urban environments.

Race and ethnicity have also been linked to an increased risk of T2DM. African Americans, Hispanics, Native Americans, Asian Americans, and Pacific Islanders have a higher incidence of type 2 diabetes compared to Caucasian populations.

While advancing age is a well-known risk factor for T2DM, the number of children being diagnosed with type 2 diabetes is also on the rise. These children are often obese, sedentary, and have a positive family history of the disease [32].

Women with a history of gestational diabetes or those who have given birth to a macrosomic infant (weighing over 4 kg) are at increased risk of developing prediabetes or overt T2DM. Moreover, polycystic ovary syndrome (PCOS) has been identified as a risk factor for type 2 diabetes mellitus [33,34].

## 4. Prevalence of Diabetes Mellitus

Diabetes mellitus is a leading chronic condition of modern society, yet estimating its true global prevalence remains challenging due to inconsistent diagnostic criteria [35]. Since the 1990s, DM rates have climbed sharply: from 366 million cases in 2011 to a projected 552 million by 2030—a 51% rise over 19 years [36]. Global reports cover all forms (type 1, type 2, gestational, and insulin-dependent/non-dependent) and complications such as retinopathy across regions [37].

Despite advances in care, life expectancy for people with DM lags behind that of the general population, primarily due to cardiovascular disease, renal failure, and infections. Type 1 diabetes (T1DM) makes up 5–10% of cases and, although it can emerge at any age, is most common in childhood and adolescence. The WHO’s DIAMOND study (children ≤14 years, year 2000) found incidence under 1 per 100,000/year in China and South America, yet over 20 per 100,000/year in Northern Europe (peaking at 36.5 in Finland), with intermediate rates in France and Italy (~8) and half of Europeans between 5 and 10 per 100,000/year. Incidence is rising in societies undergoing rapid change [38].

Type 2 diabetes (T2DM) is increasingly diagnosed in youths; school-age rates climb ~3% annually, and preschool rates ~5%. Overall, cases are set to surge in developing nations due to population growth, aging, poor diet, obesity, and inactivity. By 2030, most DM patients in wealthy countries will be ≥65, whereas those in low- and middle-income countries will predominantly be 45–64 years old. T2DM prevalence is the highest in Pacific Islands, moderate in India and the U.S., and lower in Russia; about 2–6% of Western European and North American populations are affected [39,40,41].

Urbanization, sedentary lifestyles, stress, and excess weight have doubled DM diagnoses over three decades. Projections once estimated >360 million global cases by 2030 [42]. Central (“android”) obesity, present in ~80% of T2DM patients, is a critical risk factor; waist-to-hip ratio correlates with diabetes incidence since Vague’s 1956 findings [43,44]. As of 2022, WHO reports that 43% of adults are overweight and 16% are obese worldwide, with ~35 million obese children under five in 2024 [45].

In Romania, adult DM prevalence exceeds 8%, totaling over one million people [46]. In 2011, nearly 800,000 Romanians had DM (67% urban), 14.5% were insulin-dependent, and 2651 juvenile cases were recorded (61% urban), with an age distribution of 3% (0–14 yrs), 62% (15–64 yrs), and 35% (>64 yrs). Recent estimates indicate that 1.7 million Romanians have DM and 3 million have prediabetes and are at high risk without preventive action [6,47].

By 2010, type 2 DM accounted for 90 % of the 285 million global diabetes cases, about 6% of adults—predominantly in developed nations [48,49].

## 5. Classification of Diabetes Mellitus

The etiological classification of diabetes mellitus, based on associated metabolic disturbances, allows for the differentiation of several distinct types: type 1, type 2, type 3 (resulting from specific mechanisms or underlying diseases), and type 4 (gestational diabetes) (Table 2).

Type 1 diabetes mellitus is characterized by destructive lesions of pancreatic β-cells, arising either through autoimmune mechanisms or from unknown causes [50].

**Figure 1:**
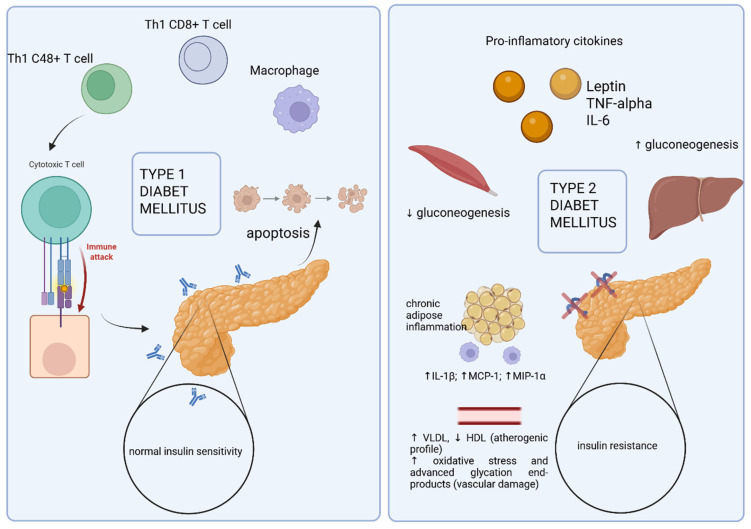
Comparative mechanisms in the development of type 1 and type 2 diabetes mellitus: immune-mediated β-cell destruction versus cytokine-induced insulin resistance (https://app.biorender.com/illustrations/682dfe31f26acbac3cfcc195, accessed on 1 May 2025).

Type 2 diabetes mellitus is defined by the coexistence of two pathophysiological features: diminished insulin secretion and reduced sensitivity of target tissues to insulin, commonly referred to as insulin resistance (Figure 1) [51].

Obesity, defined by a body mass index (BMI) greater than 30, along with metabolic syndrome, represents a significant risk factor that increases the incidence and precedes the onset of type 2 diabetes mellitus [52]. Type 3 diabetes includes two subcategories: diabetes resulting from specific genetic mutations and diabetes associated with pathological states or other diseases [53]. Gestational diabetes is defined as an impairment in glucose metabolism (glucose intolerance) that is first diagnosed during pregnancy. In certain cases, this condition may reflect previously undiagnosed preexisting diabetes, in which case it is not classified as gestational diabetes. Gestational diabetes complicates approximately 3–10% of pregnancies. Women diagnosed with gestational diabetes require long-term postnatal monitoring, often for years, due to the increased risk of developing type 2 diabetes later in life. Careful glycemic control during pregnancy is crucial, as it supports normal fetal development and significantly reduces the risk of congenital malformations and perinatal mortality [54]. Based on plasma glucose levels and glucose metabolism status, individuals may be categorized as having normal glycemia, prediabetes, or overt diabetes mellitus. The diabetic stage can be further subdivided into three clinical substages: diabetes that does not require insulin, diabetes that requires insulin for glycemic control, and diabetes that requires insulin for survival. These substages may vary depending on the progression or improvement of metabolic processes, whether wild-growing or in response to appropriate therapeutic intervention (Table 3) [55].

The classification and diagnostic criteria for diabetes mellitus were first developed in 1979 and 1980 by the National Diabetes Data Group (USA) and the Expert Committee on Diabetes Mellitus of the World Health Organization. In parallel, the American Diabetes Association (ADA) issued a report to reassess the criteria for diagnosing and classifying diabetes. The terminology was clarified, and the proposed classification system has since been widely accepted in the medical and scientific literature [49]. The etiologic classification of diabetes, initially proposed by the ADA in 1997 and endorsed by the World Health Organization (WHO) in 1999–2000, has undergone several refinements, with the most recent updates incorporated in the ADA Standards of Care in Diabetes—2024 and the WHO 2022 classification, which remain the internationally recognized frameworks for clinical and research purposes.

**Table 2:** Etiologic classification of disorders of glucose metabolism.

I. Type 1 Diabetes Mellitus (β-cell destruction, usually resulting in absolute insulin deficiency) A. Autoimmune—The autoimmune process is marked by the presence of specific autoantibodies, including islet cell antibodies, insulin autoantibodies, anti-glutamic acid decarboxylase antibodies (GAD65), and antibodies against tyrosine phosphatases IA-2 and IA-2β. B. Idiopathic—The mechanisms responsible for β-cell destruction are unknown. Patients exhibit permanent insulinopenia and are prone to ketoacidosis, without any evidence of autoimmune involvement. This form of diabetes is more commonly observed in individuals of African or Asian descent [56].
II. Type 2 Diabetes Mellitus. This form may range from predominant insulin resistance with a relative insulin deficiency to a predominant insulin secretory defect accompanied by insulin resistance [57]
III. Other Specific Types of Diabetes Mellitus A. Genetic defects of β-cell function (MODY—Maturity Onset Diabetes of the Young): Chromosome 12, HNF-1α (MODY 3) Chromosome 7, glucokinase (MODY 2) Chromosome 20, HNF-4α (MODY 1) Chromosome 13, IPF-1 (MODY 4) Chromosome 17, HNF-1β (MODY 5) Chromosome 2, NeuroD1 (MODY 6) Mitochondrial DNA mutations Subunits of the ATP-sensitive potassium channel B. Genetic defects in insulin action: Type A insulin resistance, leprechaunism, Rabson–Mendenhall syndrome, lipoatrophic diabetes C. Diseases of the exocrine pancreas: Pancreatitis, pancreatectomy, neoplasia, cystic fibrosis, hemochromatosis, fibrocalculous pancreatopathy D. Endocrinopathies: Acromegaly, Cushing syndrome, glucagonoma, pheochromocytoma, hyperthyroidism, somatostatinoma, aldosteronoma E. Drug- or chemical-induced diabetes: Vacor (a rodenticide), intravenous pentamidine (irreversibly destroys pancreatic β-cells), nicotinic acid, glucocorticoids, thyroid hormones, diazoxide, β-adrenergic agonists, thiazides, phenytoin (Dilantin), α-interferon F. Infections: Congenital rubella, cytomegalovirus G. Uncommon forms of immune-mediated diabetes: Stiff-man syndrome, insulin receptor autoantibodies H. Other genetic syndromes associated with diabetes: Down syndrome, Klinefelter syndrome, Turner syndrome, Wolfram syndrome, Friedreich ataxia, Huntington chorea, Laurence–Moon–Biedl syndrome, myotonic dystrophy, porphyria, Prader–Willi syndrome [58]
IV. Gestational Diabetes Mellitus (GDM) Carbohydrate intolerance of variable severity with onset or first recognition during pregnancy. In some cases, this may reflect previously undiagnosed pre-existing diabetes rather than true gestational diabetes. Women diagnosed with GDM require long-term follow-up after delivery [59]
V. Prediabetes This category refers to blood glucose abnormalities that are less severe than overt diabetes, but still associated with increased risk of progression and cardiovascular complications. A. Impaired Fasting Glucose (IFG): Fasting plasma glucose levels between 110 and 125 mg/dL. B. Impaired Glucose Tolerance (IGT): Two-hour plasma glucose levels during an oral glucose tolerance test between 140 and 199 mg/dL. Individuals with IFG and/or IGT are at high risk of developing type 2 diabetes, and their cardiovascular risk is elevated—comparable to that of individuals with overt diabetes [60]

**Table 3:** Categories of increased risk for diabetes (prediabetes) based on blood glucose and HbA1c values [61]. For all three tests, the risk is continuous, extending below the lower limit of the interval and becoming disproportionately high at the extreme values.

Tests Used	Reference Ranges for Prediabetes	Interpretation
FPG (fasting plasma glucose)—no carbohydrate intake 8 h prior	100 mg/dL (5.6 mmol/L) to 125 mg/dL (6.9 mmol/L)	Impaired fasting glucose
Two-hour plasma glucose after 75 g oral glucose (OGTT—oral glucose tolerance test)	140 mg/dL (7.8 mmol/L) to 199 mg/dL (11.0 mmol/L)	Impaired glucose tolerance
HbA1C	5.7–6.4%	Prediabetes

## 6. Traditionally Used Herbal Products in the Treatment of Diabetes Mellitus and Its Complications

### Key Representatives and Mechanisms of Action

**Figure 2:**
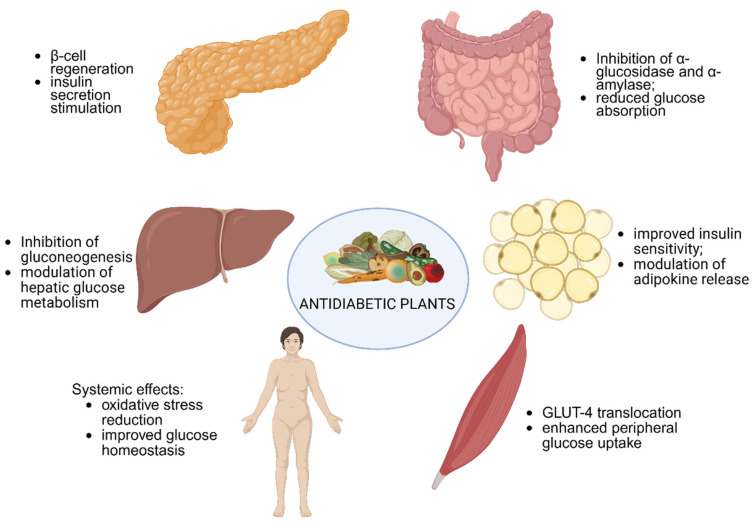
Bioactive compounds from medicinal plants exert antidiabetic effects through multiple pathways, including pancreatic β-cell stimulation and regeneration, inhibition of intestinal carbohydrate-digesting enzymes, modulation of hepatic glucose production, enhancement of peripheral insulin sensitivity, and oxidative stress reduction. Created with Biorender https://app.biorender.com/illustrations/682df40ad1bac00b3718670b, accessed on 30 April 2025.

Medicinal plants exert their antidiabetic effects through a wide range of physiological mechanisms that mirror or complement those of conventional pharmacological agents. One of the key pathways involves the regeneration and stimulation of pancreatic β-cells, ultimately leading to increased insulin production and secretion. These secretagogue-like actions are particularly valuable in early-stage diabetes, where residual β-cell function can still be preserved or enhanced. In addition to stimulating endogenous insulin secretion, many phytochemicals also display insulin-like (insulinomimetic) effects. These compounds can directly activate insulin receptors, acting as functional agonists that mimic the hormone activity and further stimulate β-cell responsiveness. Such dual mechanisms contribute not only to improved insulin levels but also to more effective glucose clearance from circulation. Another important therapeutic pathway is the reduction in intestinal glucose absorption, achieved through the inhibition of carbohydrate-digesting enzymes such as α-glucosidase and α-amylase. By slowing the breakdown of complex carbohydrates, these plant-derived compounds help attenuate postprandial glycemic spikes. Similarly, the inhibition of renal glucose reabsorption contributes to enhanced glycemic control by promoting glucose excretion via the urine, an effect functionally analogous to that of SGLT2 inhibitors. On a systemic level, phytocompounds may also exert influence over hepatic glucose metabolism through the suppression of gluconeogenesis and the modulation of glycogen storage and breakdown. This effect, in concert with enhanced glucose uptake in peripheral tissues, particularly skeletal muscle and adipose tissue, supports a more balanced glycemic profile. Additionally, plant constituents have been shown to improve insulin sensitivity, a cornerstone in the management of type 2 diabetes, particularly in individuals with metabolic syndrome. Some compounds facilitate the reactivation of protein-bound insulin, restoring its biological activity and extending its physiological utility. Finally, certain plant-based molecules contribute to glycemic regulation by inhibiting insulinase, the enzyme responsible for insulin degradation, thereby prolonging insulin half-life and bioavailability in circulation. Taken together, these multifaceted actions highlight the therapeutic potential of medicinal plants as complementary agents in diabetes management, acting not only on isolated targets but across multiple interconnected metabolic pathways (Figure 2) [62].

In conjunction with synthetic antidiabetic medications, phytotherapy serves as an effective adjunct therapy in conventional hypoglycemic treatment protocols. Medicinal plants have demonstrated therapeutic efficacy in diabetes management, with scientific studies highlighting numerous bioactive compounds that offer protective and regenerative effects on insulin-producing pancreatic β-cells [63].

The main classes of plant-derived hypoglycemic compounds include polyphenolcarboxylic acids, anthocyanosides, carotenoids, essential oils, triterpenes, thioheterosides, tannins, sterols, triterpenic saponins, proanthocyanidins, bitter principles, flavonoids, and coumarins [64,65] (Table 4).

The following table provides a comprehensive overview of the 47 medicinal plants included in this review, grouped according to the experimental models and durations most commonly used in antidiabetic research. Plants were categorized based on whether they were evaluated in cell culture assays (typically 24–48 h, assessing glucose uptake, GLUT4 translocation, or insulin secretion), rapid in vitro enzyme inhibition assays (30–60 min, targeting α-glucosidase or α-amylase activity), in vivo animal studies (ranging from 2 to 8 weeks, examining effects on glycemic control, pancreatic β-cell function, or diabetes complications), or human clinical trials (weeks to months). Notably, while many plants have demonstrated promising enzyme inhibitory effects in rapid biochemical assays, only a subset has been systematically investigated in cell-based or in vivo models that better reflect physiological glucose metabolism. Furthermore, relatively few plants have been evaluated in clinical studies, underlining the need for translational research to substantiate their therapeutic potential (Table 5).

Wild-growing plants, such as *Thymus vulgaris*, *Salvia officinalis*, and *Urtica dioica*, represent native flora that grows naturally in Romanian ecosystems without the need for human intervention. These species not only reflect the rich ethnobotanical heritage of the region but also offer a sustainable source of bioactive compounds for therapeutic use. Cultivated but non-wild-growing plants, including *Glycyrrhiza glabra*, *Trigonella foenum-graecum*, and *Asparagus officinalis*, are not native to the Romanian landscape but are widely grown due to their recognized medicinal or nutritional properties. Their inclusion in local agriculture illustrates the integration of global phytomedicine into regional practices, driven by both traditional knowledge and modern phytopharmaceutical demand. Lastly, the neither wild-growing nor commonly cultivated group, which includes tropical and subtropical species such as *Momordica charantia*, *Panax quinquefolius*, and *Azadirachta indica*, are primarily of exotic origin and are not typically found in Romanian agriculture or wild flora. These plants, however, hold significant pharmacological promise and are increasingly studied for their antidiabetic potential in global research, suggesting opportunities for future cultivation under controlled conditions or use in standardized extracts (Table 6).

Among Romania’s rich wild-growing flora, *Urtica dioica* (stinging nettle) stands out as a widely accessible, traditionally used plant with robust preclinical evidence for glycemic benefits. Both animal and in vitro studies confirm its ability to enhance glucose uptake, mimic insulin activity, and reduce oxidative stress, with an α-glucosidase IC_50_ of 44.7 μg/mL for methanolic leaf extract. Nettle can be consumed as an infusion, soup, or cooked vegetable, representing a natural and sustainable dietary intervention for blood glucose management. Its widespread availability and favorable safety profile further support its role as a practical resource for diabetes prevention in Romanian communities.

Emerging evidence consistently supports the adoption of a dietary pattern rich in whole plant foods, unprocessed grains, legumes, vegetables, nuts, and moderate fruit intake as the optimal strategy for diabetes prevention and metabolic health. In Romania, adapting the traditional diet to emphasize fiber-rich foods (whole grains, beans, lentils, and leafy greens), unsaturated fats (from nuts and seeds), and moderate dairy and fish intake, while reducing refined carbohydrates, sugary drinks, and processed meats, can substantially lower the risk of developing diabetes. Such a dietary pattern aligns with both Mediterranean and Nordic dietary recommendations and is feasible using locally available ingredients. Additionally, integrating infusions or culinary use of select medicinal plants with documented antidiabetic activity, such as *Urtica dioica* (stinging nettle), *Salvia officinalis* (sage), and *Cynara scolymus* (artichoke), offers a culturally acceptable and accessible adjunct for glycemic control. Public health efforts should focus on education, seasonal plant-based diversity, and support for rural foraging and small-scale cultivation, thus leveraging Romania’s botanical heritage for metabolic health.

Table 7 synthesizes current knowledge by categorizing all 47 medicinal plants reviewed in this article according to their principal molecular targets and antidiabetic pathways, as supported by recent pharmacological research. Each plant is mapped to at least one major mechanism, including AMPK activation, PPAR-γ modulation, PI3K/Akt-mediated GLUT4 translocation, inhibition of digestive enzymes (α-glucosidase and α-amylase), insulin receptor agonism, DPP-IV and SGLT inhibition, antioxidant and anti-inflammatory activity, β-cell protection, or other relevant metabolic effects. Notably, several plants possess multi-target actions, reflecting the polypharmacological potential of botanical therapies for diabetes management [66,67,68,69,70,71,72,73,74,75,76,77,78,79,80,81,82,83,84,85,86,87,88,89,90,91,92,93,94,95,96,97,98,99,100,101,102,103,104,105,106,107,108,109,110,111,112,113,114,115,116,117,118,119,120,121,122,123,124,125,126,127,128,129,130,131,132,133,134,135,136,137,138,139,140,141,142,143,144,145,146,147,148,149,150,151,152,153,154,155].

## 7. Materials and Methods

This review was carried out as a narrative survey with systematic search elements, guided by key PRISMA recommendations. The search was performed on PubMed, Scopus, and Web of Science for publications between January 2000 and June 2025, using broad and specific search strings such as “diabetes”, “hypoglycemic plants”, “Romania”, “phytotherapy”, “antidiabetic activity”, “in vitro”, “in vivo”, and “clinical trial”. We limited inclusion to peer-reviewed, full-text articles in English or Romanian that investigated wild or cultivated medicinal plants traditionally used in Romania for glycemic control and provided phytochemical analyses (e.g., flavonoid, alkaloid, or terpenoid profiling) and/or pharmacological assessments (enzyme assays, cell-based glucose uptake studies, animal models, or human trials). Non-original reports, abstracts, commentaries, and studies lacking clear phytochemical or efficacy data were excluded. Priority was given to controlled in vivo experiments and clinical investigations, though older or foundational phytochemical studies were retained where recent data were sparse. Extracted information encompassed plant identity, part used, extraction techniques, active constituents, experimental models, and observed outcomes. The goal was to present a comprehensive, qualitative overview of the phytochemical constituents and reported antidiabetic effects of Romanian medicinal plants rather than to conduct a formal meta-analysis or bias appraisal.

## 8. Study Limitations

Most studies rely on in vitro or animal models, and human clinical trials are limited. Additionally, herbal formulations vary in composition, which may affect reproducibility and efficacy across populations

## 9. Conclusions

Our review demonstrates that many medicinal plants used in Romanian traditional medicine possess significant antidiabetic activity, as shown by numerous in vitro and in vivo studies. Plants such as *Urtica dioica*, *Silybum marianum*, and *Momordica charantia* stand out for their strong preclinical evidence and preliminary clinical results. Their mechanisms of action, including insulin-mimetic effects, stimulation of β-cell regeneration, and reduction in oxidative stress, highlight their clinical relevance and support the idea that these species could be considered as adjuncts in diabetes management.

Despite these encouraging findings, the current body of clinical evidence remains limited. Therefore, future research should prioritize the standardization of plant extracts to ensure consistency and reproducibility across studies. Moreover, there is a clear need for well-designed, randomized controlled clinical trials to validate the efficacy and safety of these promising medicinal plants in human populations. Mechanistic studies that clarify the molecular pathways involved and incorporate biomarkers of glycemic control will also be important for understanding which patient groups are most likely to benefit from phytotherapy.

Bridging the gap between traditional use and clinical application will require collaboration between ethnobotanical research, pharmacology, and clinical sciences. By focusing on these directions, we can move closer to integrating the most effective Romanian medicinal plants into evidence-based strategies for diabetes prevention and treatment.

## Figures and Tables

**Table 1:** Genetic and Environmental Factors Influencing Type 2 Diabetes Susceptibility Across Populations.

Factor/Gene (Variant)	Description	Population (s)/Effect	Reference
South Asian origin populations	Heightened sensitivity to diabetes and increased insulin resistance; protective Pro12Ala PPARγ polymorphism in Caucasians shows no protection in Indians	Indian descent vs. Caucasians	[27]
Candidate genes (overall)	>50 genes associated with T2DM have been studied; results often inconsistent due to small samples, ethnic differences, environment, gene–environment interplay	Various worldwide populations	–
PPARγ Pro allele	Variant reduces insulin sensitivity and markedly increases T2DM risk; carries significant burden of cases	~98% of Europeans carry ≥1 Pro allele (~25% of Caucasian T2DM cases)	[28]
KCNJ11 (Lys allele) and ABCC8 (Ala variant)	Both encode subunits of the ATP-sensitive K^+^ channel (Kir6.2/SUR1) critical for insulin secretion; associated with T2DM	Pancreatic β-cells	[28]
CAPN10	Encodes calpain-10, a Ca^2+^-dependent protease; altered activity impairs insulin secretion; higher genetic risk in Mexican-Americans, lower in Caucasians	Mexican-American (↑ risk); Caucasian (↓ risk)	[29]
Viral infections (EBV, Coxsackie, mumps, CMV)	Proposed triggers for diabetes via direct β-cell destruction or autoimmune activation	–	–

**Table 4:** Plant-based hypoglycemic products used in Romania for the treatment of diabetes mellitus.

Plant Species (Family)	Active Compounds	Effective Concentration (In Vitro In Vivo)	IC_50_/EC_50_ (Target/Assay)	Mechanism of Action
*Dillenia indica* (*Dilleniaceae*), elephant apple	Tannins, pentacyclic triterpenic alcohols (betulin, betulinic acid, betulinic aldehyde), sterols (β-sitosterol), flavonosides, phenolic compounds	Betulinic acid (approx. 0.3% DW; tested as part of methanolic fruit extract at 100–400 μg/mL, in vitro)	α-glucosidase IC_50_: 30.75 μg/mL (methanolic fruit extract)	Similar to glibenclamide; has beneficial effects on the histopathological changes in the pancreas, liver, and kidneys [66,67]
*Momordica charantia* (*Cucurbitaceae*), bitter melon	Polypeptide-P, gurmarin (“plant insulin”), bitter principles (charantin)	mcIRBP peptide (natural content in fresh fruit: approx. 0.5–1.2 mg/g; tested in vitro/in vivo); cucurbitane triterpenoids (content not specified; tested at 10 μM, in vitro)	Charantin α-glucosidase IC_50_: 10.8 μg/mL *(isolated compound)*; mcIRBP EC_50_: 0.96 μg/mL	Action begins 30–60 min after oral administration with peak activity at 4 h; mimics bovine insulin, has antioxidant properties, regenerates β-cells, stimulates insulin secretion (sulfonylurea-like mechanism), increases glycogenogenesis, insulin-like activity [68,69]
*Glycyrrhiza glabra* (*Fabaceae*), licorice	Sterols, flavones, tannins, enzymes, saponins, volatile oils	Glycyrrhizin (natural content in root: approx. 2–9% DW; tested as part of ethanolic root extract at 10–100 μM, in vitro)	α-glucosidase IC_50_: 12.8 μg/mL (ethanolic root extract)	Acts as a PPAR agonist, improving hyperinsulinemia [70]
*Trigonella foenum-graecum* (*Fabaceae*), fenugreek	Sterols (lecithin, phytic acid, other phytosterols), bitter substances, volatile oil, tannins, saponins, coumarins	Diosgenin (natural content in seeds: approx. 0.1–0.3%; tested at 8–19 mM, in vitro); ethanolic seed extract (galactomannan: 30–50%; tested at 3–11 μg/mL, in vitro)	Diosgenin EC_50_ (GLUT4): 8 mM (pure compound); α-glucosidase IC_50_: 30.15 μg/mL (ethanolic seed extract)	Stimulates glucose transport; regulates glycolysis, gluconeogenesis, and fatty acid synthesis; reduces oxidative stress associated with hyperglycemia; and delays the progression of diabetic retinopathy and neuropathy [71,72,73]
*Pueraria lobata* (*Fabaceae*), kudzu	Sterols, coumarins, saponins, isoflavones	Puerarin (natural content in root: approx. 3–9%; tested at 100 μM, in vitro; as part of ethanolic root extract)	α-glucosidase IC_50_: 45.4 μg/mL (ethanolic root extract)	Restores secretory function of β-cells, enhances insulin secretion, inhibits glucose absorption; isoflavones act as PPAR agonists, block IL-12 synthesis, inhibit TH-1 differentiation; prevent and delay T2DM and cardiovascular complications [74,75]
*Phaseolus vulgaris* (*Fabaceae*), common bean	Sulfur-containing amino acids, alkaloids, anthocyanins, flavonoids, saponins, tannins, terpenoids	Phaseolamin (natural content in extract: approx. 0.5–2%; tested as part of aqueous seed extract at 100 μg/mL, in vitro	α-amylase IC_50_: 44.2 μg/mL (aqueous seed extract)	Stimulates insulin secretion, increases glucose tolerance; potency similar to tolbutamide [76]
*Pimpinella anisum* (Apiaceae), anise	Volatile oil (80–90% anethole, methyl chavicol or isomethylchavicol, small amounts of anisic ketones and aldehydes)	Anethole (main component, essential oil; tested as methanolic seed extract at 50–200 μg/mL, in vitro)	α-glucosidase IC_50_: ~67 μg/mL (methanolic seed extract)	Increases glutathione-S-transferase activity, has antioxidant effect, reduces cholesterol and triglyceride levels [77,78]
*Apium graveolens* (*Apiaceae*), celery	Sedanenolides, neocnidilide, neophytadiene, essential oil (limonene, β-selinene, nerolidol, α-selinene, β-pinene, carvone, β-myrcene)	Phthalides and apigenin (main actives; natural content not specified; tested as ethanolic seed extract at 100–400 μg/mL, in vitro)	Not available (no IC_50_ for glucose uptake); α-glucosidase IC_50_: ~80 μg/mL (ethanolic seed extract)	Induces insulin receptor phosphorylation Promotes GLUT-4 translocation (in muscle and adipose cells) Inhibits gene expression involved in adipogenesis Enhances peripheral glucose utilization [79,80]
*Daucus carota* (*Apiaceae*), carrot	Mineral salts, carotenoids, vitamins (C, B-complex, folic acid), fibers (cellulose and lignin), acids (glutamic, succinic, lactic, glycolic), polyphenolcarboxylic acids (caffeic acid), anthocyanins	Carotenoids, caffeic acid derivatives (natural content: β-carotene ~7–14 mg/100g; extract tested at 100–400 μg/mL, in vitro)	α-glucosidase IC_50_: 75 μg/mL (aqueous root extract)	Improves glucose tolerance Inhibit enzymes of glucose metabolism [81,82]
*Thymus vulgaris* (Lamiaceae), thyme	Volatile oils (thymol, p-cymene, borneol, geraniol, carvacrol, linalool, bornyl acetate, α-pinene), saponins, ursolic acid, oleanolic acid, caffeic acid, flavonoids (luteolin, luteolin-7-glycoside), sterols, waxes, triterpenes, bitter principles	Thymol, carvacrol (essential oil; tested as ethanolic aerial part extract at 50–200 μg/mL, in vitro)	α-glucosidase IC_50_: 49.8 μg/mL (ethanolic aerial part extract)	Reduces hyperinsulinemia, increases SOD (superoxide dismutase) concentration, counteracts oxidative effects, preventing diabetic complications [83,84]
*Lavandula angustifolia* (*Lamiaceae*), lavender	Volatile oil (linalyl acetate, linalyl butyrate, geraniol, free linalool, linalyl valerate, borneol, α-pinene), coumarins, caryophyllene, tannins, bitter principles	Linalool (main in oil; natural content not specified; hydroalcoholic flower extract tested at 50–200 μg/mL, in vitro)	α-glucosidase IC_50_: 72.8 μg/mL (hydroalcoholic flower extract)	Stimulates glucose uptake in muscle cell cultures, increases insulin secretion via a sulfonylurea-like mechanism [85,86]
*Salvia officinalis* (*Lamiaceae*), sage	Essential oil, thujone, α- and β-pinene, camphor, borneol, cineole, tannins, sitosterols, estrogen-like substances, bitter principles (picrosalvin), nicotinic acid, caffeic acid, fumaric acid, resins, vitamins B1 and C, mineral salts	Thujone, rosmarinic acid (thujone ~30–50% oil; extract tested at 50–200 μg/mL, in vitro)	α-glucosidase IC_50_: 59.4 μg/mL (ethanolic leaf extract)	Stimulates insulin production and secretion; increases glucose utilization in tissues similarly to metformin; inhibits glucose absorption [87,88]
*Phyllanthus emblica* (*Phyllanthaceae*), Indian gooseberry	Tannins (gallic acid, ellagic acid), norsesquiterpenoids, flavonosides	Gallic/ellagic acid (main actives; total phenolics ~50–100 mg GAE/g; extract tested at 50–200 μg/mL, in vitro)	α-glucosidase IC_50_: 46.2 μg/mL (ethanolic fruit extract)	Inhibits neuropathic pain by modulating oxidative stress, nitrite levels, cytokines (IL-1β, TGF-β1); used as a strong antioxidant and immunomodulator; inhibits α-amylase and α-glucosidase; reduces severity of acute pancreatitis and promotes pancreas repair; lowers cholesterol and triglycerides, improves liver function via ALT normalization [89,90]
*Asparagus racemosus* (*Asparagaceae*), shatavari	Tannins, saponins (shatavarins A and B, filiasparoside C, asparanin A), isoflavones, satavarin (a glycoside of glucose, rhamnose, and sarsapogenin), vanillin, coniferin, sarsaponin	Shatavarin IV (main saponin; content ~0.1–0.2% root; extract tested at 50–200 μg/mL, in vitro)	α-glucosidase IC_50_: 85 μg/mL (ethanolic root extract)	Tannic acid induces insulin receptor phosphorylation, mediates GLUT-4 translocation; inhibits adipogenesis-related gene expression; prevents diabetic nephropathy; used for polydipsia in diabetes insipidus [91,92]
*Azadirachta indica* (*Meliaceae*), Neem	Bitter compounds: nimbin, nimbinin, and nimbidin. Leaves contain quercetin, beta-sitosterol, the diterpenoids mahogany and nimbogone, vitamins (A, E, C, riboflavin, and niacin), and minerals (Se, Zn, Cu, Mg, Cr)	Nimbin, nimbidin (main actives; extract tested at 50–200 μg/mL, in vitro)	α-amylase IC_50_: 52.4 μg/mL (aqueous leaf extract)	Decreases glucose absorption Antioxidant properties, inhibits α-amylase and α-glucosidase Inhibits SGLUT-1 activity Increases activity of glucose-6-phosphate dehydrogenase Exerts insulin-like effect Increases levels of superoxide dismutase, catalase, glutathione peroxidase, and glutathione transferase Hypoglycemic effect comparable to glibenclamide [93,94]
*Garcinia cambogia* (*Clusiaceae*), garcinia	Tartaric, citric, and phosphoric acids; two poly-isoprenylated benzophenones; mangostin derivatives of camboginol and cambogin	Hydroxycitric acid (HCA; content in fruit rind ~16–20%; extract tested at 100–500 μg/mL, in vitro)	α-amylase IC_50_: 60.5 μg/mL (aqueous fruit extract)	The mentioned acids suppress lipogenesis by inhibiting citrate lyase, which assists in converting excess carbohydrates into fat Reduces triglycerides and cholesterol; resin confers satiety Main constituent, hydroxycitric acid, reduces appetite and increases fat oxidation [95,96]
*Cinnamomum zeylanicum* (*Lauraceae*), cinnamon tree	Diterpenes, essential oil rich in eugenol, cinnamaldehyde, cinnamyl acetate, cinnamic alcohol, and 2-hydroxycinnamaldehyde, coumarins, terpenes, tannins, proanthocyanidins	Cinnamaldehyde, polyphenols (content in bark oil: cinnamaldehyde 65–80%; extract tested at 10–100 μg/mL, in vitro)	α-glucosidase IC_50_: 16.3 μg/mL (aqueous bark extract)	Mimics insulin action (via tannic acid) Stimulates glycogen synthesis Contains glutathione and flavonoids (MHCP—methylhydroxychalcone polymer), increasing adipose tissue sensitivity to insulin [97,98]
*Panax quinquefolius* (*Araliaceae*), American ginseng	Saponozide, saponins, tannins, bitter principles, vitamins A, B1, B2, B5, B6, B12, D3, E, folic acid, nicotinamide, mucilage, waxes, Zn, K, Fe, Si	Ginsenosides (content in root: 3–5%; extract tested at 10–100 μg/mL, in vitro)	α-glucosidase IC_50_: 38.2 μg/mL (ethanolic root extract)	Stabilizes blood glucose levels by increasing tissue insulin sensitivity Polypeptides exert hypoglycemic action and stimulate hepatic glycogen synthesis Strong antioxidant activity Increases HDL-cholesterol plasma fraction [99,100]
*Ginkgo biloba* (*Ginkgoaceae*)**,** maidenhair tree	Diterpenes (ginkgolides A, B, C and ginkgolide J), sesquiterpenes (bilobalide); leaves contain flavonols (kaempferol, quercetin, isorhamnetin), biflavones (bilobetin, ginkgetin, isoginkgetin), catechins, proanthocyanidins, sterols, 6-hydroxymurenic acid	Ginkgolide/flavone (leaf extract; content: ginkgolide 0.5–1% DW; tested at 50–200 μg/mL, in vitro)	α-glucosidase IC_50_: 41.3 μg/mL (ethanolic leaf extract)	Improves blood circulation and prevents complications Prevents insulin resistance [101,102,103]
*Silybum marianum* (*Asteraceae*), milk thistle	Saponins, essential oil; fruits contain silymarin (silibinin, silidianin, silicristin), betaine hydrochloride, amino acids (L-cysteine, glycine, glutamic acid, D-2-aminobutyric acid, D-leucine, tyramine), lipids (3–4%), polyhydroxyphenylchromones, fumaric acid	Silymarin (fruit/seed extract; content: silymarin 1.5–3% seeds; tested at 50–200 μg/mL, in vitro)	α-glucosidase IC_50_: 32.4 μg/mL (methanolic seed extract)	Stimulates glucose transport Regulates glycolysis, gluconeogenesis, and fatty acid synthesis Reduces oxidative stress related to hyperglycemia Delays diabetic retinopathy and neuropathy Increases glucose tolerance Reduces AGE and ALE formation Silibinin improves β-cell viability and may serve as a therapeutic agent for type 2 diabetes [104,105]
*Acorus calamus* (*Araceae*), sweet flag	Volatile oil (1.5–3.5%) containing asarone, azaril aldehyde, methyl isoeugenol, linalool, sesquiterpenes, α-pinene, camphene, camphor, eugenol, tannins, bitter substances, resins, mineral salts	β-asarone (rhizome oil; content: 1–3% in oil; extract tested at 25–100 μg/mL, in vitro)	α-glucosidase IC_50_: 28.1 μg/mL (ethanolic rhizome extract)	Increases insulin release and secretion similarly to gliclazide; inhibits α-glucosidase and improves insulin resistance; inhibits preadipocyte differentiation into adipocytes; β-asarone attenuates ERK1/2 phosphorylation involved in early adipogenesis [106]
*Achillea millefolium* (*Asteraceae*), yarrow	Volatile oil, chamazulene, azulenes, asarone, proazulenes, cineole, borneol, pinene, limonene, caryophyllene, achilleine, achilleic acid, organic acids (formic, valeric), tannins	Chamazulene, apigenin (extract tested at 50–200 μg/mL, in vitro)	α-glucosidase IC_50_: 61.2 μg/mL (methanolic aerial part extract)	Regenerates β-pancreatic cells; hypolipidemic effect [107,108]
*Arctium lappa* (*Asteraceae*), burdock	Arctiin, essential oil, flavonoids, inulin, palmitic acid, caffeic acid, stigmasterol, sitosterol, bitter substances, carotenoids, mineral salts	Arctiin, inulin (inulin content: 30–50% root DW; extract tested at 100–400 μg/mL, in vitro)	α-glucosidase IC_50_: 37.4 μg/mL (aqueous root extract)	β-sitosterol-D glucopyranoside inhibits α-glucosidase; inulin improves glucose tolerance [109,110]
*Artemisia absinthium* (*Asteraceae*), wormwood	Essential oil (0.5%) with myrcene, α-pinene, thujone, nerol, camphor, limonene, phellandrene, β-caryophyllene, sesquiterpene lactones (artabsin, absinthin)	Thujone, absinthin (oil: thujone 30–50%; extract tested at 50–200 μg/mL, in vitro)	α-glucosidase IC_50_: 43.1 μg/mL (methanolic aerial part extract)	Hypoglycemic effects similar to metformin; stimulates glycogenogenesis [111,112]
*Cichorium intybus* (*Asteraceae*), chicory	Inulin, cichorin, chicoric acid, volatile oil, flavonoids, anthocyanins, bitter triterpenes (lactucopicrin), tannins, mineral salts	Inulin (root content: 15–20% DW; methanolic root extract tested at 100–400 μg/mL, in vitro)	α-glucosidase IC_50_: 45.5 μg/mL (methanolic root extract)	Improves glucose tolerance; reduces hepatic glucose-6-phosphatase activity [113,114]
*Cynara scolymus* (*Asteraceae*), artichoke	Polyphenols (caffeic acid, chlorogenic acid, cynarin), flavones, potassium, and magnesium salts	Cynarin, chlorogenic acid (leaf content: chlorogenic acid 1–2% DW; extract tested at 50–200 μg/mL, in vitro)	α-glucosidase IC_50_: 36.5 μg/mL (methanolic leaf extract)	Insulin-mimetic, hypolipidemic, antioxidant effects [115,116]
*Taraxacum officinale* (*Asteraceae*), dandelion	Flavones (rutoside, hyperoside, quercetol), hydroxycinnamic acid derivatives (caffeic and chlorogenic acid), catechin tannins, sterols, triterpenes, carotenoids, coumarins, mucilage	Caffeic acid, inulin (root: inulin 15–25% DW; extract tested at 100–400 μg/mL, in vitro)	α-glucosidase IC_50_: 42.7 μg/mL (aqueous root extract)	Tannins reduce amylase activity by chelating calcium; hypolipidemic effects [117,118]
*Sambucus nigra* (*Caprifoliaceae*), elderberry	Anthocyanins, essential oil, quercetin derivatives, cyanogenic glycoside (sambunigrin), mucilage, flavonosides	Anthocyanins, flavonols (flower content: total anthocyanins ~200–400 mg/100g DW; extract tested at 50–200 μg/mL, in vitro)	α-glucosidase IC_50_: 55.1 μg/mL (aqueous flower extract)	Insulin-like effect; increases insulin secretion and release [119,120]
*Spinacia oleracea* (*Chenopodiaceae*), spinach	Flavonoids (quercetin, myricetin, kaempferol, apigenin, luteolin, spinacetin), phenolic acids (ferulic, coumaric), carotenoids, vitamins (A, E, C, K, folic acid), minerals	Quercetin, kaempferol (leaf content: quercetin 10–30 mg/100 g FW; extract tested at 50–200 μg/mL, in vitro)	α-glucosidase IC_50_: 62.3 μg/mL (aqueous leaf extract)	Potentiates insulin and protects β-pancreatic cells from oxidative damage similar to glibenclamide [121,122]
*Juglans regia* (*Juglandaceae*), walnut	Riboflavin, niacin, vitamin C, ellagic tannins, inositol, juglone, essential oil. The pericarp contains juglone (5-hydroxy-1,4-naphthoquinone), tannins, etheric oil, chlorophylls, starch, pectins, organic acids	Juglone, ellagitannins (leaf content: juglone ~20–50 mg/100 g; extract tested at 50–200 μg/mL, in vitro)	α-glucosidase IC_50_: 35.2 μg/mL (methanolic leaf extract)	Increases tissue sensitivity to insulin, induces phosphorylation of insulin receptors, involved in GLUT-4 translocation, and inhibits the expression of certain genes [123,124]
*Aloe vera*, (*Liliaceae*), aloe	Aloe-emodin, aloin A and B, aloe-emodin, chrysophanol (free or glycosidic), resins (10–20%), essential oil in small quantities, mineral salts	Aloin, aloe-emodin (gel content: aloin 0.1–0.4%; extract tested at 50–200 μg/mL, in vitro)	α-glucosidase IC_50_: 50.9 μg/mL (aqueous gel extract)	Reduces blood glucose in type 2 diabetes with insulin-like activity. Lowers blood lipids and triglycerides Enhances insulin effects and protects β-pancreatic cells from oxidative damage Hypoglycemic action similar to metformin Increases GLUT-4 mRNA synthesis Lowers TC, LDL, TG, and VLDL; increases hepatic glycogen; inhibits lipogenesis [125,126]
*Asparagus officinalis* (*Liliaceae*), asparagus	Asparagine, lipids, carbohydrates, phytohormones, enzymes, sterols, cellulose, mineral salts	Asparagine, saponins (root: asparagine ~0.03%; extract at 50–200 μg/mL, in vitro)	α-glucosidase IC_50_: 69.3 μg/mL (methanolic root extract)	Potentiates insulin and protects β-pancreatic cells from oxidative damage [127,128]
*Allium cepa* (*Liliaceae*), onion	Cycloalliin, methylalliin, propylalliin, cepaenes, flavonoid derivatives (quercetin and kaempferol glycosides), saponins, amines, enzymes	Quercetin, alliin (bulb: quercetin ~10–30 mg/100 g FW; extract at 50–200 μg/mL, in vitro)	α-glucosidase IC_50_: 40.1 μg/mL (aqueous bulb extract)	Hypoglycemic effect similar to glibenclamide and insulin [129,130]
*Allium sativum* (*Liliaceae*), garlic	Sulfur compounds; flavonosides; vitamins (A, B1, B2, C); phytosterol; glycerides of palmitic, stearic, oleic, linoleic, and myristic acids; allicin; steroid derivatives (erubosides)	Allicin, alliin (bulb: allicin ~0.1–0.5%; extract at 50–200 μg/mL, in vitro)	α-glucosidase IC_50_: 28.9 μg/mL (ethanolic bulb extract)	Hypoglycemic and hypolipidemic effects, similar to glibenclamide and insulin [131,132]
*Viscum album* (*Loranthaceae*), mistletoe	Triterpenic saponins, oleanolic acid derivatives, viscotoxin, viscol, amines (choline, acetylcholine), β-phenylethylamine, lipids, glycosidic substances	Oleanolic acid glycosides, viscotoxins (leaf: oleanolic acid ~0.2–0.4%; extract at 50–200 μg/mL, in vitro)	α-glucosidase IC_50_: 58.4 μg/mL (aqueous leaf extract)	Increases insulin secretion and peripheral glucose utilization; hypoglycemic effect comparable to glibenclamide [133,134]
*Morus alba / nigra* (*Moraceae*), mulberry	Citric, aspartic, folic, and folinic acids, volatile compounds, β-carotene, tannins, phenolic compounds, alkaloids, anthocyanins, minerals, vitamins C, B2, B3	DNJ (1-deoxynojirimycin, leaf: 0.1–0.2%; extract at 50–200 μg/mL, in vitro)	α-glucosidase IC_50_: 32.1 μg/mL (methanolic leaf extract)	Stimulates cellular glucose uptake; insulin-mimetic effect [135,136]
*Alchemilla vulgaris* (*Rosaceae*), lady mantle	Ellagic tannins, polyphenolcarboxylic acids (chlorogenic acid), saponins, flavonoids, ellagic and luteic acid, fatty compounds (stearic and palmitic acids), phytosterols, mineral salts	Ellagitannins, chlorogenic acid (aerial part: ellagitannins ~1%; extract at 50–200 μg/mL, in vitro)	α-glucosidase IC_50_: 57.6 μg/mL (aqueous aerial part extract)	Improves glucose tolerance; anorexigenic effect [137,138]
*Fragaria ananassa* (*Rosaceae*), strawberry	Fragarol; oily substances; citric, malic, and ascorbic acids; anthocyanins; citrol; polyphenols; vitamins A, B, C	Anthocyanins, ellagic acid (fruit: anthocyanins 20–50 mg/100 g FW; extract at 50–200 μg/mL, in vitro)	α-glucosidase IC_50_: 47.9 μg/mL (methanolic fruit extract)	Inhibits α-glucosidase; antioxidant effect [139,140]
*Rosa canina* (*Rosaceae*), rosehip	Carotenoids, terpenoids, anthocyanins, vitamins C, B1, B2, PP, K	Ascorbic acid, flavonoids (fruit: ascorbic acid 0.3–0.7%; extract at 50–200 μg/mL, in vitro)	α-glucosidase IC_50_: 53.2 μg/mL (methanolic fruit extract)	Stimulates insulin secretion [141,142]
*Agrimonia eupatoria* (*Rosaceae*), common agrimony	Catechin-type tannins, gallotannins, ellagitannins, free quercetin, hyperin, rutin, apigenin and luteolin glycosides, bitter substances, traces of essential oil, ursolic acid, organic acids, mucilage, coumarins, vitamins (C, K), triterpenes, fatty acids, flavonoids, saponins	Catechin, ellagitannins (aerial part: ellagitannins ~0.8%; extract at 50–200 μg/mL, in vitro)	α-glucosidase IC_50_: 50.2 μg/mL (methanolic aerial part extract)	Stimulates glucose uptake in cultured muscle cells Increases insulin secretion via sulfonylurea-like mechanism [143,144]
*Citrus aurantium* (*Rutaceae*), bitter orange	Citric and malic acids, calcium and potassium citrate, vitamins A, B1, B2, B3, D, E, PP, essential oil (limonene, nerolidol, terpineol, farnesol, pinene, phellandrene), flavonoids	Hesperidin, synephrine (peel: hesperidin ~0.2–0.5%; extract at 50–200 μg/mL, in vitro)	α-glucosidase IC_50_: 54.5 μg/mL (ethanolic fruit peel extract)	Reduces insulin resistance, lowers LDL-cholesterol and triglycerides, antioxidant activity [145,146]
*Lycopersicon esculentum* (*Solanaceae*), tomato	Flavonoids, lycopene, organic acids (malic, pectic, citric), vitamins A, B1, B2, B6, C, PP, E, K, β-carotene, minerals	Lycopene, β-carotene (fruit: lycopene 2–5 mg/100g FW; extract at 50–200 μg/mL, in vitro)	α-glucosidase IC_50_: 60.1 μg/mL (methanolic fruit extract)	Stimulates insulin secretion, improves insulin resistance [147,148]
*Urtica dioica* (*Urticaceae*), stinging nettle	Polyphenols, amino acids, sterols, essential oil, sitosterols, ursolic acid, vitamins C, B2, K, chlorophylls, protoporphyrin, β-carotene, alkaloids	Phenolic compounds, sterols (leaf: total phenolics 20–40 mg/g DW; extract at 50–200 μg/mL, in vitro)	α-glucosidase IC_50_: 44.7 μg/mL (methanolic leaf extract)	Insulin-mimetic effect, reduces LDL-cholesterol [149]
*Vitis vinifera* (*Vitaceae*), grapevine	Polyphenols, resveratrol, anthocyanins, flavonosides, tartaric and malic acid, tannins, minerals, vitamins A, C, E	Resveratrol, proanthocyanidins (seed: proanthocyanidins 5–10%; extract at 50–200 μg/mL, in vitro)	α-glucosidase IC_50_: 38.6 μg/mL (methanolic seed extract)	Antioxidant, reduces oxidative stress, insulin-like effect, lowers glucose absorption, regenerates β-pancreatic cells [150,151]
*Vaccinium arctostaphylos* (*Ericaceae*), bearberry	Flavonoids (quercetin), tannins	Quercetin, arbutin (leaf: arbutin 5–7% DW; extract at 50–200 μg/mL, in vitro)	α-amylase IC_50_: 42.2 μg/mL (aqueous leaf extract)	Inhibits α-amylase [152,153]
*Hippophae rhamnoides* (*Elaeagnaceae*), sea buckthorn	Carotenoids, flavonoids, proanthocyanidins, catechin tannins, triterpenic acids, vitamin C	Carotenoids, flavonoids, vitamin C (fruit: carotenoids 3–8 mg/100g FW; extract at 50–200 μg/mL, in vitro)	α-glucosidase IC_50_: 48.3 μg/mL (ethanolic fruit extract)	Inhibits α-glucosidase [154,155]

IC_50_—half maximal inhibitory concentration; FW—fresh weight; DW—dry weight; n.a.—not available.

**Table 5:** Distribution of the antidiabetic medicinal plants by experimental model and duration in antidiabetic research.

Experiment Duration/Model	Plants Tested in This Model
Cell culture (glucose uptake/GLUT4, 24–48 h, insulin secretion)	*Momordica charantia*, *Trigonella foenum-graecum*, *Pueraria lobata*, *Glycyrrhiza glabra*, *Phaseolus vulgaris*, *Agrimonia eupatoria*, *Spinacia oleracea*, *Juglans regia*, (*unele studii pentru Allium cepa*, *Allium sativum*, *Morus alba/nigra*, *Rosa canina*, *Apium graveolens*, *Silybum marianum*, *Cichorium intybus*, *Asparagus officinalis*, *Aloe vera*, *Urtica dioica*, *Vitis vinifera*)
Enzyme inhibition (α-glucosidase/α-amylase, 30–60 min, in vitro enzyme assay)	*Dillenia indica*, *Glycyrrhiza glabra*, *Phaseolus vulgaris*, *Trigonella foenum-graecum*, *Pueraria lobata*, *Apium graveolens*, *Daucus carota*, *Thymus vulgaris*, *Lavandula angustifolia*, *Salvia officinalis*, *Pimpinella anisum*, *Silybum marianum*, *Acorus calamus*, *Achillea millefolium*, *Arctium lappa*, *Artemisia absinthium*, *Cichorium intybus*, *Cynara scolymus*, *Taraxacum officinale*, *Sambucus nigra*, *Spinacia oleracea*, *Juglans regia*, *Urtica dioica*, *Vitis vinifera*, *Vaccinium arctostaphylos*, *Hippophae rhamnoides*, *Alchemilla vulgaris*, *Fragaria ananassa*, *Rosa canina*, *Citrus aurantium*, *Lycopersicon esculentum*, *Morus alba/nigra*, *Allium cepa*, *Allium sativum*, *Viscum album*, *Asparagus racemosus*, *Azadirachta indica*, *Garcinia cambogia*, *Cinnamomum zeylanicum*, *Panax quinquefolius*, *Phyllanthus emblica*
Animal studies (in vivo, 2–8 weeks, effect on glycemia, β-cell, complications, etc.)	*Momordica charantia*, *Trigonella foenum-graecum*, *Glycyrrhiza glabra*, *Silybum marianum*, *Achillea millefolium*, *Cynara scolymus*, *Taraxacum officinale*, *Sambucus nigra*, *Juglans regia*, *Urtica dioica*, *Rosa canina*, *Citrus aurantium*, *Hippophae rhamnoides*, *Allium cepa*, *Allium sativum*, *Viscum album*, *Azadirachta indica*, *Panax quinquefolius*, *Phyllanthus emblica*, *Cichorium intybus*, *Pueraria lobata*, *Spinacia oleracea*, *Alchemilla vulgaris*, *Fragaria ananassa*, *Agrimonia eupatoria*, *Asparagus racemosus*, *Morus alba/nigra*, *Acorus calamus*, *Daucus carota*, *Apium graveolens*, *Arctium lappa*, *Lavandula angustifolia*, *Salvia officinalis*, *Thymus vulgaris*, *Vaccinium arctostaphylos*, *Aloe vera*, *Vitis vinifera*, *Garcinia cambogia*, *Dillenia indica*, *Phyllanthus emblica*, *Pimpinella anisum*, *Thymus vulgaris*
Human/clinical studies (weeks–months, when available)	*Momordica charantia*, *Trigonella foenum-graecum*, *Silybum marianum*, *Cinnamomum zeylanicum*, *Allium sativum*, *Vitis vinifera*, *Salvia officinalis*, *Panax quinquefolius*, *Morus alba/nigra*, *Aloe vera*, *Glycyrrhiza glabra*

**Table 6:** Ethnobotanical categorization of antidiabetic plants based on their occurrence in Romania.

Wild-Growing Plants in Romania (Native Wild Flora)	Not Wild-Growing but Cultivated in Romania:	Neither Wild-Growing nor Commonly Cultivated in Romania:
➢ *Apium graveolens* ➢ *Daucus carota* ➢ *Thymus vulgaris* ➢ *Lavandula angustifolia* ➢ *Salvia officinalis* ➢ *Achillea millefolium* ➢ *Arctium lappa* ➢ *Artemisia absinthium* ➢ *Silybum marianum* ➢ *Cichorium intybus* ➢ *Taraxacum officinale* ➢ *Sambucus nigra* ➢ *Juglans regia* ➢ *Acorus calamus* ➢ *Viscum album* ➢ *Morus alba/nigra* ➢ *Alchemilla vulgaris* ➢ *Rosa canina* ➢ *Agrimonia eupatoria* ➢ *Urtica dioica* ➢ *Vaccinium arctostaphylos* ➢ *Hippophae rhamnoides*	➢ *Glycyrrhiza glabra* ➢ *Trigonella foenum-graecum* ➢ *Phaseolus vulgaris* ➢ *Spinacia oleracea* ➢ *Asparagus officinalis* ➢ *Fragaria ananassa* ➢ *Citrus aurantium* ➢ *Lycopersicon esculentum* ➢ *Pimpinella anisum* ➢ *Apium graveolens* ➢ *Allium cepa* ➢ *Allium sativum* ➢ *Vitis vinifera*	➢ *Dillenia indica* ➢ *Momordica charantia* ➢ *Pueraria lobata* ➢ *Phyllanthus emblica* ➢ *Asparagus racemosus* ➢ *Azadirachta indica* ➢ *Garcinia cambogia* ➢ *Cinnamomum zeylanicum* ➢ *Panax quinquefolius* ➢ *Ginkgo biloba* ➢ *Aloe vera*

**Table 7:** Molecular target and pathway distribution of antidiabetic medicinal plants.

Molecular Target/Pathway	Medicinal Plants (Main Bioactive Compounds)
AMPK activation	*Momordica charantia* (charantin), *Silybum marianum* (silymarin), *Vitis vinifera* (resveratrol), *Viscum album*, *Arctium lappa* (oleanolic acid), *Spinacia oleracea* (quercetin), *Pueraria lobata* (puerarin), *Glycyrrhiza glabra* (possibly via anti-inflam.), *Lavandula angustifolia* (rosmarinic acid)
PPAR-γ modulation	*Glycyrrhiza glabra* (glycyrrhizin), *Pueraria lobata* (puerarin), *Viscum album*, *Silybum marianum*, *Vitis vinifera*
PI3K/Akt → GLUT4 translocation	*Trigonella foenum-graecum* (diosgenin), *Momordica charantia* (mcIRBP), *Glycyrrhiza glabra*, *Pueraria lobata*, *Allium cepa* (quercetin), *Spinacia oleracea*
α-glucosidase inhibition	*Morus alba/nigra* (DNJ), *Phaseolus vulgaris* (phaseolamin), *Cinnamomum zeylanicum* (cinnamaldehyde), *Dillenia indica*, *Arctium lappa* (arctiin), *Vaccinium arctostaphylos*, *Salvia officinalis*, *Apium graveolens*, *Cichorium intybus*, *Silybum marianum*, *Acorus calamus*, *Achillea millefolium*, *Cynara scolymus*, *Taraxacum officinale*, *Sambucus nigra*, *Allium cepa*, *Urtica dioica*, *Fragaria ananassa*, *Rosa canina*, *Hippophae rhamnoides*, *Allium sativum*, *Azadirachta indica*, *Asparagus racemosus*, *Morus alba*, *Citrus aurantium*, *Phyllanthus emblica*, *Vitis vinifera*, *Garcinia cambogia*, *Panax quinquefolius*, *Aloe vera*, *Vitis vinifera*
α-amylase inhibition	*Dillenia indica*, *Phaseolus vulgaris*, *Taraxacum officinale*, *Garcinia cambogia*, *Vaccinium arctostaphylos*, *Azadirachta indica*
Insulin receptor agonism/similar effect	*Momordica charantia* (mcIRBP, polypeptide-p), *Allium sativum* (allicin), *Urtica dioica*, *Salvia officinalis*, *Citrus aurantium*
DPP-IV inhibition	*Trigonella foenum-graecum* (trigonelline), *Glycyrrhiza glabra*, *Salvia officinalis*, *Citrus aurantium*
SGLT inhibition	*Trigonella foenum-graecum* (trigonelline), *Morus alba* (DNJ), *Citrus aurantium*
Antioxidant/Anti-inflammatory	*Vitis vinifera*, *Sambucus nigra*, *Rosa canina*, *Spinacia oleracea*, *Lavandula angustifolia*, *Achillea millefolium*, *Thymus vulgaris*, *Fragaria ananassa*, *Phyllanthus emblica*, *Asparagus officinalis*, *Artemisia absinthium*
β-cell regeneration/insulin secretion	*Pueraria lobata*, *Sambucus nigra*, *Allium sativum*, *Salvia officinalis*, *Citrus aurantium*, *Agrimonia eupatoria*, *Artemisia absinthium*
Miscellaneous (hepatic glucose regulation, lipid metabolism)	*Cichorium intybus* (hepatic effect), *Garcinia cambogia* (ATP citrate lyase), *Cynara scolymus* (lipid/glucose), *Aloe vera* (GLUT4), *Hippophae rhamnoides*

## Data Availability

Data are contained within the article.
